# The effectiveness and reliability of autologous hematopoietic stem cell transplantation following chemotherapy in managing malignant lymphoma: a meta-analysis

**DOI:** 10.1007/s12672-025-01876-x

**Published:** 2025-02-13

**Authors:** Hend Ahmed, Ahmed S. Shafiey, Mohamed E. A. Abdelrahim

**Affiliations:** 1 Faculty of Pharmacy, Beni-Suef University, Beni-Suef, Egypt; 2 Clinical Pharmacy Department, Faculty of Pharmacy, Beni-Suef University, Beni-Suef, Egypt

**Keywords:** Autologous hematopoietic stem cell transplantation, Chemotherapy, Malignant, Lymphoma

## Abstract

**Background:**

Autologous hematopoietic stem cell transplantation (AHSCT) is a valuable treatment option for several hematological malignancies, particularly in relapsed or refractory cases. Autologous hematopoietic stem cell transplantation (AHSCT) is effective in improving survival rates in selected patients, particularly those with aggressive lymphomas and multiple myeloma. Studies suggest AHSCT may outperform alternative therapies, but ongoing research is essential to refine patient selection. Many patients enjoy prolonged remission and improved quality of life, indicating the need for long-term follow-up to assess late effects and overall survival. This work aimed to establish meta-analysis to methodically evaluate the safety and effectiveness of autologous stem cell therapy (AHSCT) in the management of malignant lymphoma following high-dose chemotherapy and to produce reliable findings that may serve as a foundation for clinical application and reference.

**Methods:**

A systematic literature search was performed from February 2017 to August 2024, and malignant lymphoma was identified as the study subjects' diagnosis. The experimental group was identified as AHSCT afterwards high-dose chemotherapy, while the control group underwent standard chemotherapy (with no restrictions on the chemotherapy regimen). The outcome indicators were progression-free survival (PFS), complete remission rate (complete response (CR) + partial response (PR)), and overall survival (OS).

**Results:**

Fifteen literature pieces in all, consisting of 1229 subjects in the control group and 896 subjects in the experimental group, were included. Conventional chemotherapy (chemotherapy regimen not limited) was the intervention strategy used in the control group. The odds ratio (OR) was 2.23, with a 95% confidence interval (CI) of [1.54, 3.22], Z = 4.25; P < 0.0001, indicating that the groups differed in overall survival and progression-free survival rates. Similarly, the progression-free survival rate was 2.70, with a 95% CI of 1.86–3.92, Z = 4.25; P < 0.0001, and overall survival was 2.23.

**Conclusions:**

Patients with malignant lymphoma who receive chemotherapy can substantially extend their overall survival and progression-free survival rates with AHSCT treatment.

## Introduction

Lymphoma is a form of cancer that arises from lymphocytes are an essential t immune system component found in the lymphatic system and are the origin of a specific type of cancer which is lymphoma. There are two different types of the disease: Hodgkin lymphoma (HL) and non-Hodgkin lymphoma (NHL). Hodgkin lymphoma can be distinguished by Reed-Sternberg cells, this type has a more predetermined therapeutic response [1]. As commonly referenced in oncology literature and guidelines from reputable organizations, non-Hodgkin lymphoma accounts for ~ 85% of lymphoma cases and includes a wide array of subtypes [2]. Managing NHL shows complexity as it necessitates a multidisciplinary approach and often close collaboration between oncologists, hematologists, and other healthcare professionals. Treating non-Hodgkin lymphoma (NHL) can be challenging due to its various subtypes, each with specific characteristics and treatment responses, demanding personalized approaches. The aggressiveness of certain forms requires immediate intervention, while others do not, complicating management strategies, individual patient factors, such as age and overall health, further influence treatment options. Furthermore, the side effects of therapies like chemotherapy and radiation can significantly impact the quality of life, and the risk of relapse presents ongoing challenges [3].

Autologous hematopoietic stem cell transplantation (AHSCT) holds significant value in managing non-Hodgkin lymphoma (NHL), particularly for patients with relapsed or refractory disease. This approach involves obtaining the patient's stem cells and high-dose chemotherapy to eradicate cancer cells. AHSCT can lead to durable remission and improved survival rates, as it allows for the recovery of the immune system while minimizing the risk of graft-versus-host disease associated with allogeneic transplants. Limited clinical randomized controlled trial data resulted in different conclusions [4]. This meta-analysis investigated the effectiveness of AHSCT in managing malignant lymphoma after high-dose chemotherapy to address its drawbacks and give a reference for clinical application.

## Methods

The study completed here followed the meta-analysis of studies in the epidemiology statement, [5] following an established protocol.

### Study selection

Studies comprised stated statistical measures of relationship (odds ratio [OR] with 95% confidence intervals [CIs]) measuring the safety and efficacy of AHSCT in subjects with malignant lymphoma.

**Fig. 1:**
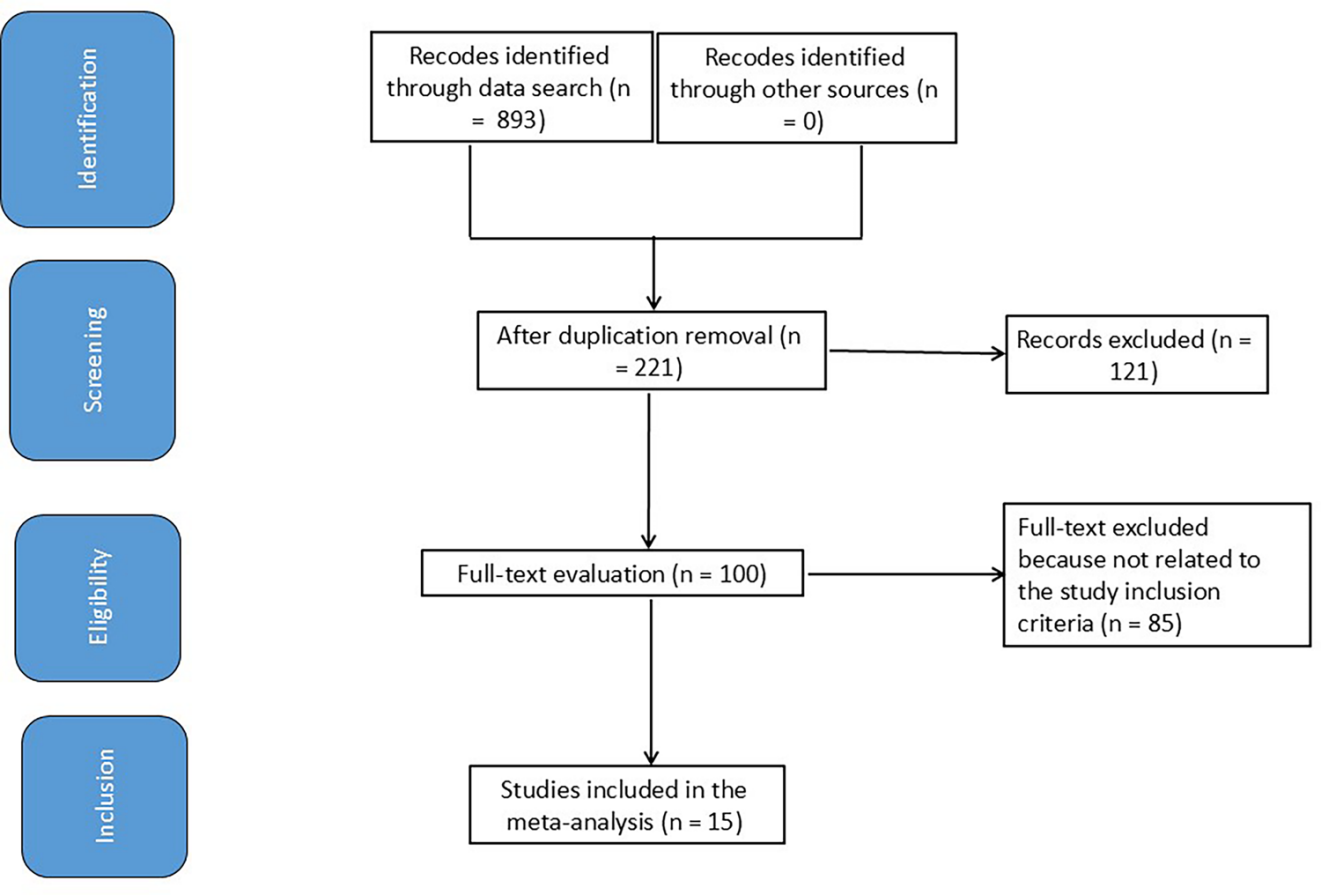
Diagram of the study process

Only human studies in any language were selected. Neither the study type nor size impacted the inclusion. Studies excluded were commentary, review articles, and articles that did not provide a degree of association. Figure 1 shows the whole study process.

The articles were included in our meta-analysis when the next inclusion criteria were met:

1. The study was a prospective study or retrospective.
2. Randomized control trials and non-randomized control trials.
3. The target population is subjects with malignant lymphoma
4. The experimental group was treated with AHSCT after high-dose chemotherapy, and the control group was treated with conventional chemotherapy.
5. The study comprised comparisons between the safety and efficacy of AHSCT.

The exclusion criteria were:

1. Studies that did not compare AHSCT to control.
2. Studies with diseases other than malignant lymphoma.
3. Studies did not concentrate on the effect of comparative results.

### Identification

A search protocol strategies were organized according to the PICOS principle [6], as follows: P (population): subjects with malignant lymphoma; I (intervention/exposure): AHSCT after high dose chemotherapy; C (comparison): safety and efficacy of HCT-AHSCT in subjects with malignant lymphoma; O (outcome): overall survival (OS), complete remission rate [complete response (CR) + partial response (PR)], and event-free survival (EFS); and S (study design): no restriction [7]. First, a systematic survey searched OVID, Embase, Cochrane Library, PubMed, and Google Scholar, from February 2017 to August 2024, using the search terms included malignant lymphoma, autologous hematopoietic stem cell transplantation, AHSCT, high-dose chemotherapy, etc. as shown in Table 1. Selected studies were collected in an EndNote file, duplicates were omitted, and the title and abstracts were reviewed to remove studies that did not report the association between the safety and efficacy of HCT-AHSCT in subjects with malignant lymphoma based on the previously mentioned exclusion and inclusion criteria. The remaining articles were revised for associated information.

**Table 1:** Database Search Strategy for inclusion of examinations

Database	Search strategy
Google Scholar	# 1"malignant lymphoma"[MeSH Terms] OR "malignant lymphoma" #2 "autologous hematopoietic stem cell transplantation"[MeSH Terms] OR "AHSCT" #3 "chemotherapy"[MeSH Terms] OR "chemotherapy" #4 "overall survival" OR "complete remission" OR "event-free survival"
Embase	#1 (adverse effects):ti,ab,kw OR (quality of life):ti,ab,kw OR (clinical efficacy):ti,ab,kw (Word variations have been searched) #2 (hematologic toxicity): ti,ab,kw OR (autologous hematopoietic stem cell transplantation):ti,ab,kw OR (chemotherapy):ti,ab,kw OR (malignant lymphoma):ti,ab,kw (Word variations have been searched) #3 #1 AND #2
Cochrane Library	#1 (adverse effects):ti,ab,kw OR (quality of life):ti,ab,kw OR (clinical efficacy):ti,ab,kw (Word variations have been searched) #2 (hematologic toxicity): ti,ab,kw OR (autologous hematopoietic stem cell transplantation):ti,ab,kw OR (chemotherapy):ti,ab,kw OR (malignant lymphoma):ti,ab,kw (Word variations have been searched) #3 #1 AND #2
Pubmed	# 1"malignant lymphoma"[MeSH Terms] OR "malignant lymphoma" #2 "autologous hematopoietic stem cell transplantation"[MeSH Terms] OR "AHSCT" #3 "chemotherapy"[MeSH Terms] OR "chemotherapy" #4 "overall survival" OR "complete remission" OR "event-free survival"
OVID	# 1"malignant lymphoma"[MeSH Terms] OR "malignant lymphoma" #2 "autologous hematopoietic stem cell transplantation"[MeSH Terms] OR "AHSCT" #3 "chemotherapy"[MeSH Terms] OR "chemotherapy" #4 "overall survival" OR "complete remission" OR "event-free survival"

### Screening

**Fig. 2:**
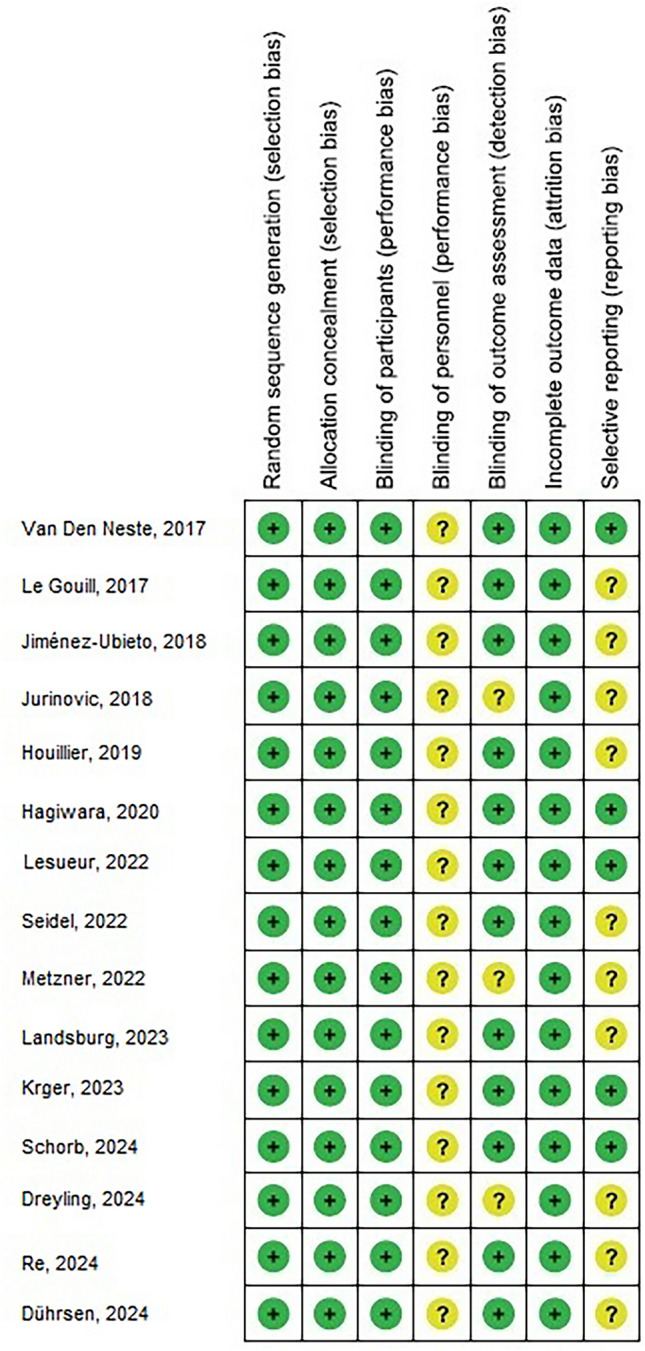
Cochrane risk-of-bias tool (RoB 2)

Data were abbreviated based on the following; study-associated and subject-associated features in a homogeneous form. The primary author's last name, study period, publication year, country, the study region, and design of the study; type of the population, total number and subjects’ number, demographic data, and clinical and treatment features; the evaluation period associated with measurement, quantitative method and qualitative method of assessment, source of information, and outcomes assessment; and statistical analysis The calculation method used OR as the effect size and 95% CI was used to express the result. If a study fit for inclusion based upon the principles mentioned above, data were extracted individually by two authors. In case of discrepancy, the corresponding author made a decision. The data were extracted independently from studies that contained a variety of data. Each study's bias risk was evaluated by two authors who separately assessed the chosen studies' methodological quality. We used the “risk of bias tool" from the RoB 2: A revised Cochrane risk-of-bias tool for randomized trials to evaluate methodological quality and output the bias risk chart, as shown in Fig. 2 [8]. In terms of the evaluation criteria, each study was valued and allocated to one of the next three risks of bias: low: if all quality criteria were met; unclear or moderate: if one or more of the quality criteria were partially or unclearly met or high: if one or more of the criteria were not met at all., or not included. Any discrepancies were addressed by a reassessment of the original article.

### Eligibility

The main result concentrated on measuring the safety and efficacy of HCT-AHSCT in subjects with malignant lymphoma. Evaluation of the safety and efficacy of HCT-AHSCT in subjects with malignant lymphoma was extracted forming a summary.

### Inclusion

Sensitivity analyses were restricted only to studies showing the relationship between the safety and efficacy of AHSCT in subjects with malignant lymphoma. For subcategory and sensitivity analysis, the effect of AHSCT compared to control was used.

### Statistical analysis

The method of calculation utilized was OR, while the result was expressed using effect size and 95% CI. OR = (ratio of exposed to non-exposed in case group)/(ratio of exposed to non-exposed in the control group). We determined the I^2^ index and the I^2^ index was alternated between 0 and 100%. When the I^2^ index was about 0%, 25%, 50%, and 75% that identifies no, low, moderate, and high heterogeneity, respectively. (6) We used the random effect if the I^2^ was > 50%; we used the fixed effect if it was < 50%. We used to stratify the original evaluation per outcome categories as described before to complete the subgroup analysis. A p-value for differences between subcategories of < 0.05 was considered statistically significant. Publication bias was evaluated quantitatively using the Egger regression test (publication bias exists if p ≥ 0.05), and qualitatively, by visual examination of funnel plots of the logarithm of odds ratios against their standard errors. (8) The whole p-values were 2-tailed. Reviewer Manager version 5.3 (The Nordic Cochrane Centre, The Cochrane Collaboration, Copenhagen, Denmark) was used to do all calculations and graphs.

## Results

A total of 893 related documents were retrieved in this study, and 221 papers were obtained after eliminating duplicates. After reading the full texts of these articles, 121 papers that did not meet the requirements were excluded. After further reading of the full text, 85 articles that did not meet the requirements were excluded.

Finally, 15 documents that met the inclusion criteria were included [9–23]. The literature retrieval and selection process are shown in Fig. 1, and Table 2 displays the basic information of the included literature which of 15 studies (between 2017 and 2024) satisfied the inclusion criteria and were included in the study.

**Table 2:** Studies characters

Study	Country	Total	Experimental	Control
Van Den Neste, 2017 [9]	United Kingdom	74	16	58
Le Gouill, 2017 [10]	Algeria	240	120	120
Jiménez‐Ubieto, 2018 [11]	Spain	68	16	52
Jurinovic, 2018 [12]	Germany	162	63	99
Houillier, 2019 [13]	France	97	44	53
Hagiwara, 2020 [14]	Japan	12	5	7
Lesueur, 2022 [15]	France	34	5	29
Seidel, 2022 [16]	Germany	181	50	131
Metzner, 2022 [17]	Germany	54	17	37
Landsburg, 2023 [18]	US	209	100	109
Kröger, 2023 [19]	Germany	41	19	22
Schorb, 2024 [20]	Germany	87	36	51
Dreyling, 2024 [21]	Germany	582	292	290
Re, 2024 [22]	Italy	88	44	44
Dührsen, 2024 [23]	Germany	196	69	127
	Total	2125	896	1229

The 15 studies included 2125 subjects with traumatic brain injury at the start of the study; 896 of them underwent AHSCT and 1229 were controlled. All studies evaluated the safety and efficacy of AHSCT in subjects with malignant lymphoma.

### Overall survival rate

**Fig. 3:**
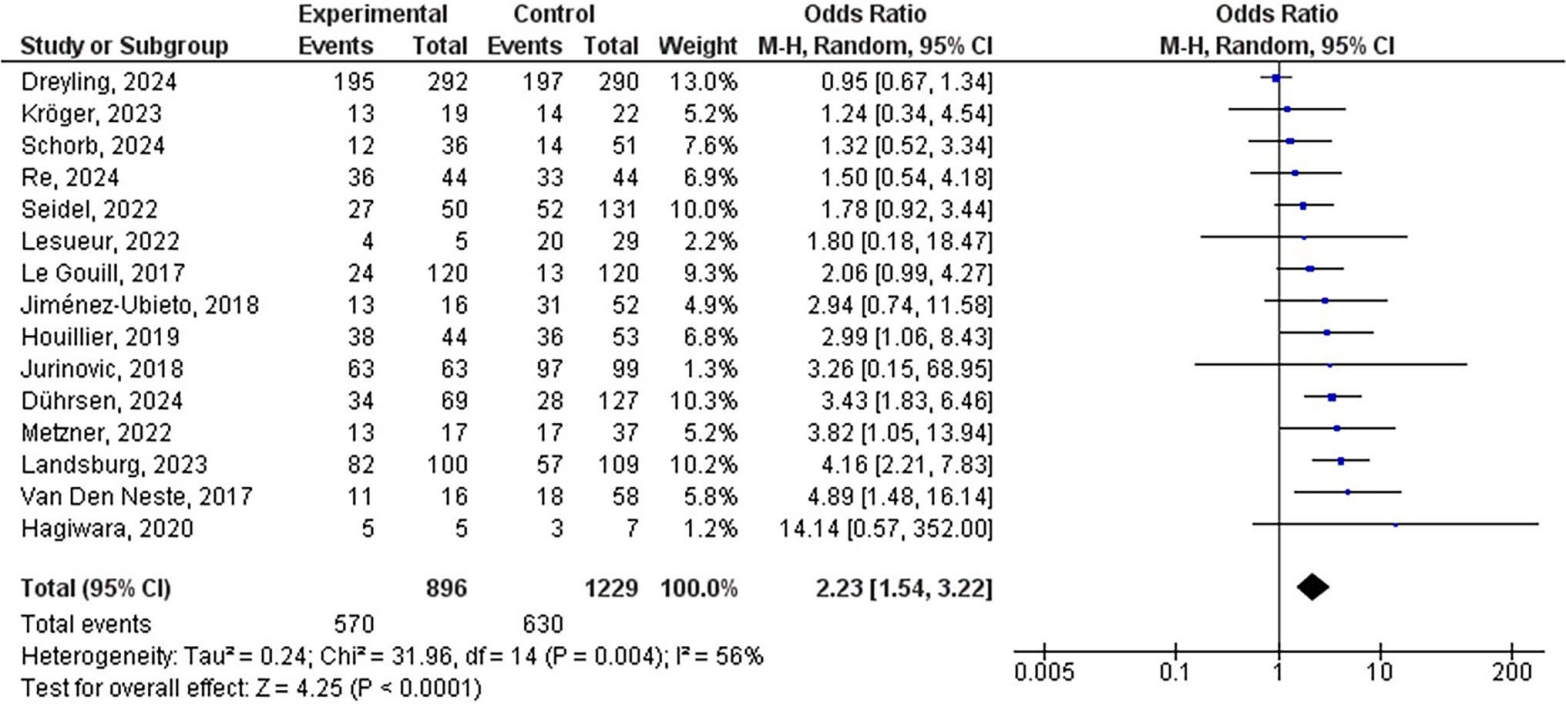
Forest plot showing the overall survival rate results of patients

The survival rates of the experimental and control groups have been evaluated using a literature review and screening of fifteen trials. Meta-analysis of the overall survival of patients receiving AHSCT (Fig. 3) was performed, and the heterogeneity analysis results showed I^2^ = 56%, so the REM was used for analysis. After a meta-analysis of the comprehensive structure model, the results showed that OR = 2.23, 95% CI: 1.54–3.22 Z = 4.25, and P < 0.0001, which indicates AHSCT significantly improved survival rates in patients with malignant lymphoma and poor chemotherapy response (P < 0.05), indicating its effectiveness and most of the data corresponded to points within the 95% CI, which indicated that the publication bias was low.

### Partial remission rates comparison

**Fig. 4:**
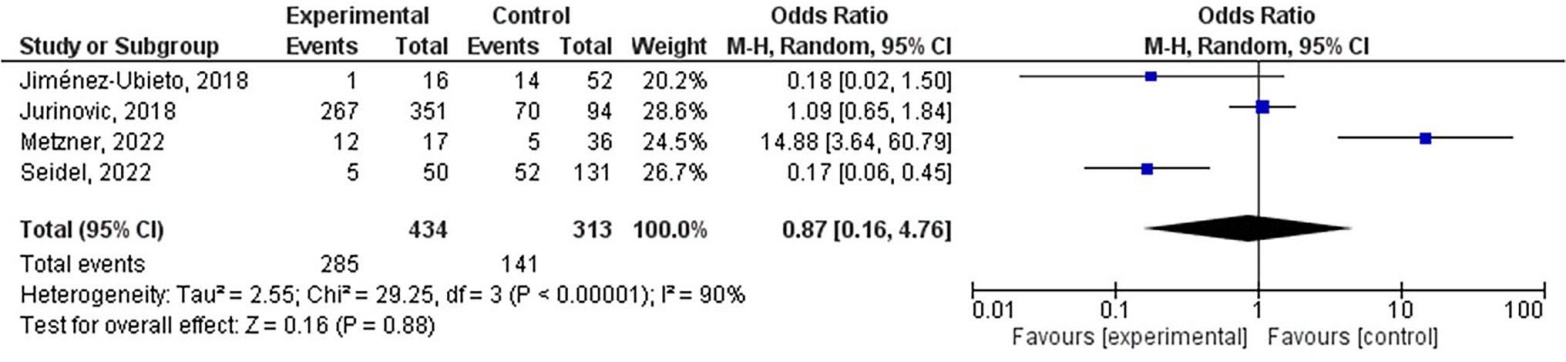
Forest diagram analysis of the partial remission rate

The rates of partial remission in both the experimental and control groups were examined through a review of the literature and analysis of four studies. A meta-analysis was conducted on the partial remission rate of patients undergoing AHSCT (Fig. 4), with heterogeneity analysis findings indicating an I^2^ statistic of 90%, leading to the utilization of the random effects model (REM) for analysis. Following a comprehensive analysis of the structural model, the findings revealed an OR of 0.87, with a 95% CI of 0.16–4.76, Z = 0.16, and P = 0.88, indicating no significant difference in partial remission rates between patients who underwent AHSCT and those who did not (P > 0.05). The majority of data points fell within the 95% CI, suggesting low publication bias.

### Complete remission rate comparison

**Fig. 5:**
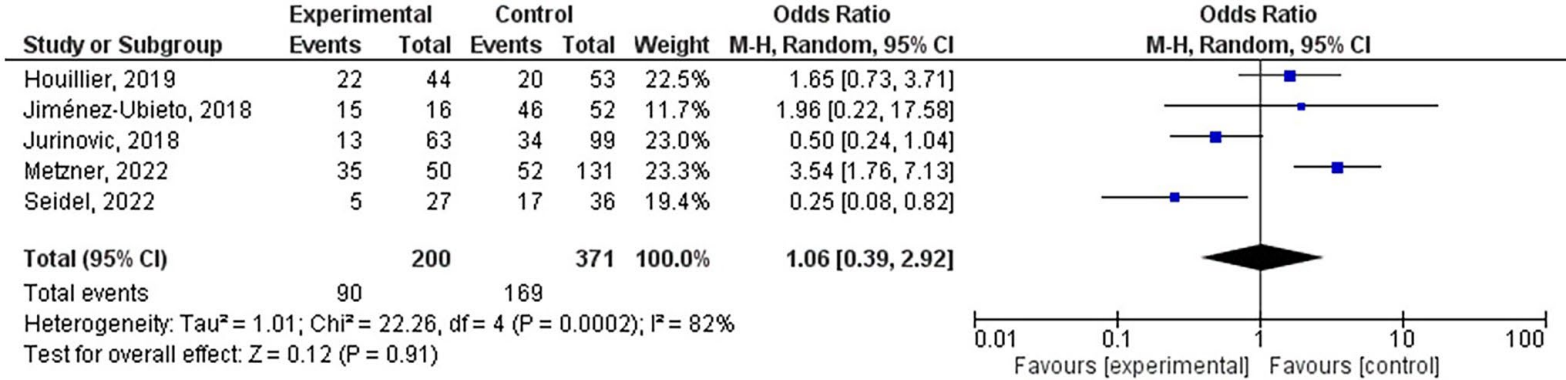
Forest diagram analysis of the complete remission rate

The rates of complete remission in both the experimental and control groups were examined by reviewing five studies in the literature. A meta-analysis was done on the full remission rate of patients who underwent AHSCT (Fig. 5), with heterogeneity results indicating I^2^ = 82%, leading to the use of REM for analysis. Upon conducting a meta-analysis on the detailed structural model, it was found that the OR was equal to 1.06, with a 95% confidence interval of 0.39–2.92, a Z-score of 0.12, and a p-value of 0.91. These findings suggest that there is no notable disparity in the rate of complete remission among patients who underwent AHSCT compared to those who did not undergo the procedure (p > 0.05).

### Progression-free survival rate comparison

**Fig. 6:**
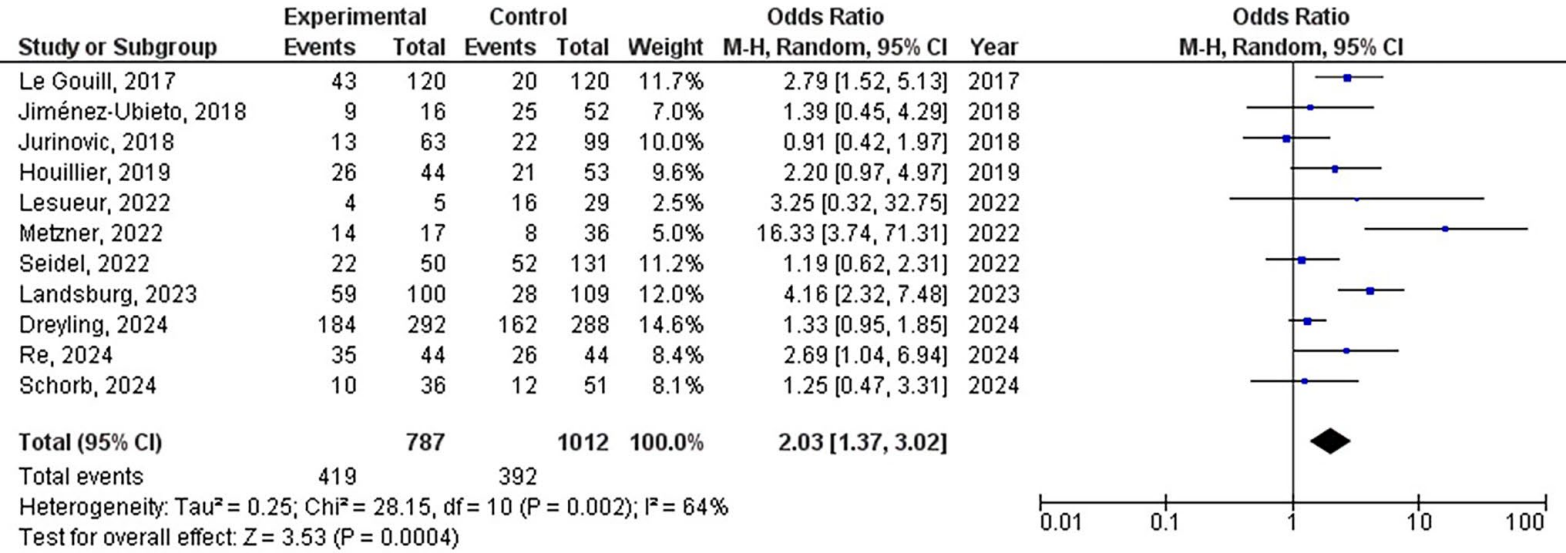
Forest diagram analysis of the progression-free survival rate

An investigation was conducted on eleven studies to compare the progression-free survival rates between the experimental and control groups. After analyzing the progression-free survival rate of patients receiving AHSCT in a meta-analysis (Fig. 6), it was found that there was a heterogeneity level of I^2^ = 64%, leading to the use of the REM for analysis. The meta-analysis of the comprehensive structure model revealed OR = 2.03, 95% CI: 1.37, 3.02, Z = 3.53, and P < 0.0001, suggesting a significant disparity in progression-free survival rates between patients who underwent AHSCT and those who did not undergo AHSCT (P < 0.05).

## Discussion

Lymphoma is a form of cancer arising from lymphocytes, important immune system components in the lymphatic system. The disease is divided into Hodgkin lymphoma (HL) and non-Hodgkin lymphoma (NHL) [24]. This disease is complex, has a high incidence rate, and is readily metastasized, making treatment more challenging. Most lymphomas respond well to chemotherapy; following conventional treatment, some individuals experience long-term survival and are cured of their disease [25]. Despite this, a lot of people continue to have poor outcomes from traditional chemotherapy, which worsens their conditions and finally results in death. To achieve more effective curative outcomes, physicians started to increase the dosage of chemotherapeutic medications in response to the difficulties above. Clinical research findings, however, demonstrated that while high-dose chemotherapy medications can destroy tumor cells, they also cause substantial harm to the patient's immune system and hematopoietic system in their bone marrow, which has a serious effect on their prognosis and survival. This led to the establishment of AHSCT technology, whose primary feature is the extraction and in vitro cryopreservation of the patient's hematopoietic stem cells. Following high-dose chemotherapy, whole-body irradiation, or total lymph node irradiation, the obtained cells are reintroduced into the patient to help in the recovery of their hematopoietic and immune systems [26]. Several clinical trials have demonstrated the effectiveness of high-dose chemotherapy in conjunction with AHSCT in the treatment of lymphoma. It can be stated that the most significant and successful lymphoma treatment option available now is AHSCT. For young patients who have relapsed or become not sensitive to chemotherapy, as well as those who are sensitive to the drug, it is a successful first-line treatment strategy. In certain cases, AHSCT is a viable therapy option for individuals who are over 65. While there is now little data to support the serious effects of using AHSCT as the first line of treatment for lymphoma, it can increase the disease's rate of control and in certain cases, lead to a patient's cure and progression-free survival. Although AHSCT is generally effective in treating lymphoma, a more thorough study is still needed to fully validate its safety, indications, and side effects. This meta-analysis reported the efficacy of using AHSCT with malignant lymphoma. However, additional studies are required to confirm these possible relations. Moreover, additional studies are required to supply a clinically meaningful difference in the outcomes in subjects with malignant lymphoma. These studies must include larger homogeneous samples. This was recommended similarly in an earlier meta-analysis study which reported a comparable effectiveness of HCT-AHSCT and control in subjects with malignant lymphoma [27].

## Limitations

There may be selection bias in this study since several selected studies were excluded from the meta-analysis. However, the excluded studies did not satisfy the inclusion criteria of our meta-analysis. The study designed to evaluate the association between the safety and efficacy of HCT-AHSCT in subjects with malignant lymphoma was based on data from earlier studies, which may result in bias persuaded by incomplete details. Eventually, this approach is still controversial. More prospective randomized trials are needed to figure out whether this approach is effective as a first-line treatment for lymphoma. While there is less information on the risks of first-line AHSCT for lymphoma treatment, it can improve disease control and even lead to disease-free survival for certain individuals. While AHSCT is effective in treating lymphoma, more research is needed to determine its safety, indications, and potential side effects.

## Conclusions

In this study, we addressed the literature related to malignant lymphoma patients treated with AHSCT after obtaining high-dose chemotherapy. A meta-analysis was carried out on fifteen articles covering various parameters such as overall survival, partial remission, complete remission, and event-free survival. It was shown that patients who got AHSCT following high-dose chemotherapy had a much higher survival rate than those who did not. These findings indicate that high-dose chemotherapy followed by autologous stem cell transplantation (AHSCT) has a good therapeutic influence on individuals for whom conventional chemotherapy was unsatisfactory so it has a greater promising potential for use in the treatment of malignant tumors.

## Data Availability

The data supporting this study's findings are not publicly available but can be shared by the corresponding author upon reasonable request. The data is currently stored in an Excel sheet.
